# Supplementary material for: Genetic, Transcriptomic, and Epigenomic Insights into Sjögren’s Disease: An Integrative Network Investigation and Immune Diseases Comparison
Source: Int J Mol Sci. 2025 May 13;26(10):4637. doi: 10.3390/ijms26104637 (PMC12111751; doi:10.3390/ijms26104637)
Supplement: Supplementary file 1 [file ijms-26-04637-s001.zip › Figures S1-S14.pdf]

Supplementary Material 2

Genetic, Transcriptomic, and Epigenomic Insights into Sjögren's Disease: An Integrative Network Investigation and Immune Diseases Comparison

Nitesh Enduru [1,2], Astrid M. Manuel [1,3] and Zhongming Zhao [1,2,*]

[1] Center for Precision Health, McWilliams School of Biomedical Informatics, The University of Texas Health Science Center at Houston, Houston, TX 77030, USA; nitesh.enduru@uth.tmc.edu (N.E.); astrid.manuel@bcm.edu (A.M.M.)
[2] Department of Epidemiology, Human Genetics and Environmental Sciences, School of Public Health, The University of Texas Health Science Center at Houston, Houston, TX 77030, USA
[3] Molecular & Human Genetics Department, Baylor College of Medicine, Houston, TX 77030, USA
* Correspondence: zhongming.zhao@uth.tmc.edu

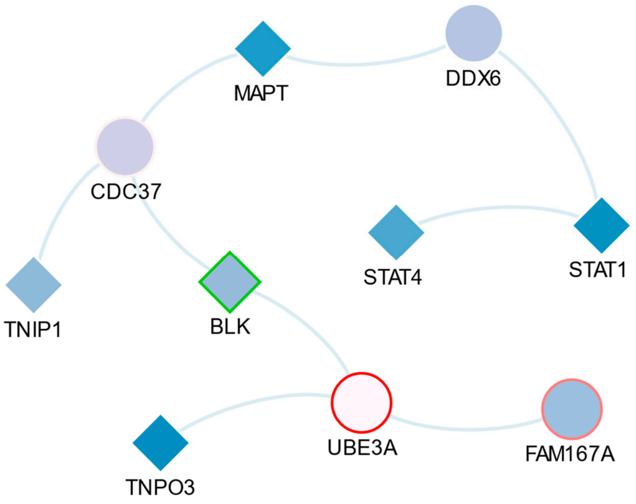

Figure S1. Protein-protein interaction of top 1 module gene (differentially methylated genes) from discovery data.

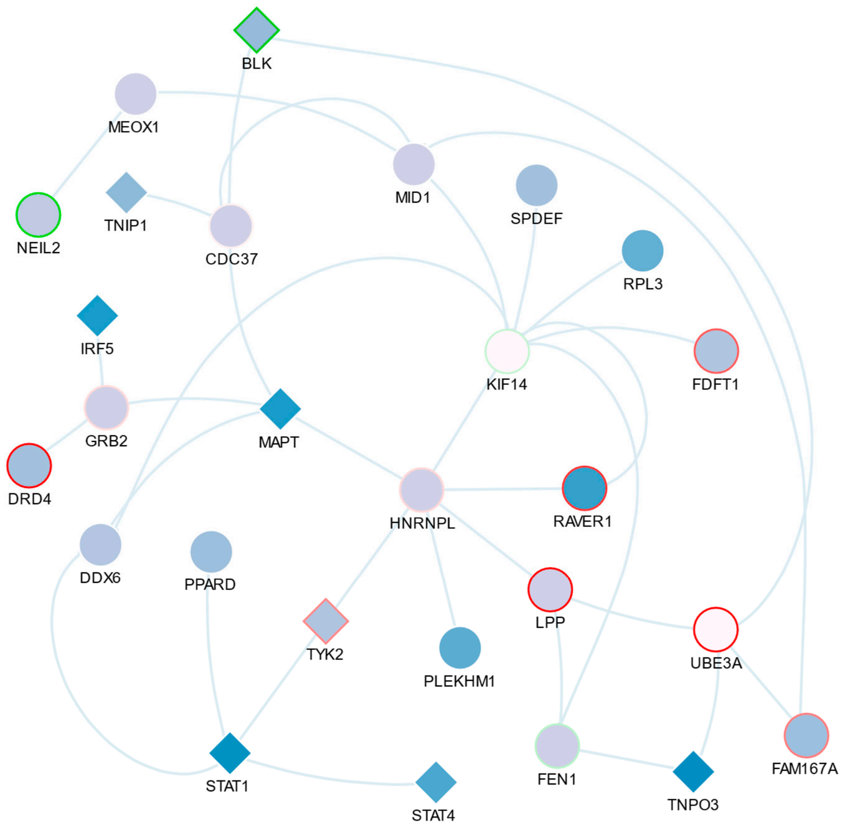

Figure S2. Protein-protein interaction of top 5 module gene (differentially methylated genes) from discovery data.

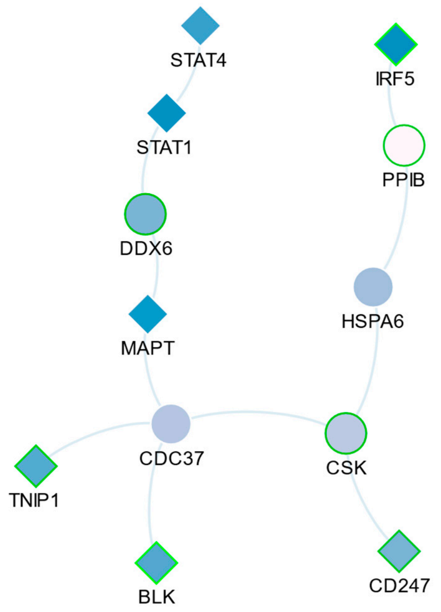

Figure S3. Protein-protein interaction of top 1 module gene (differentially methylated genes) from evaluation data.

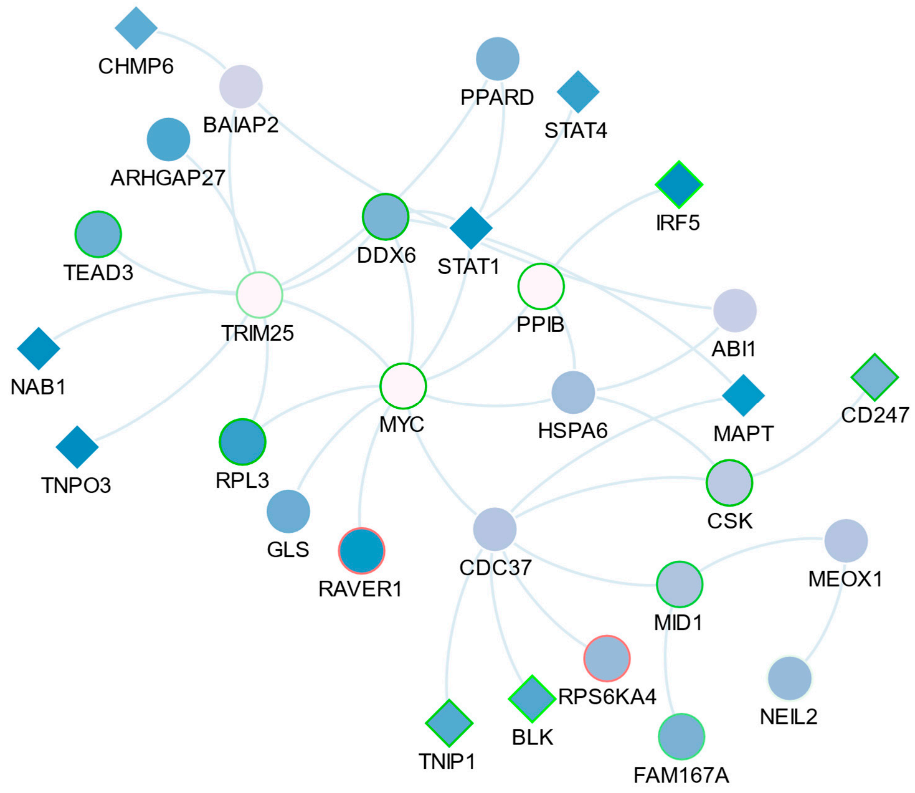

Figure S4. Protein-protein interaction of top 5 module gene (differentially methylated genes) from evaluation data.

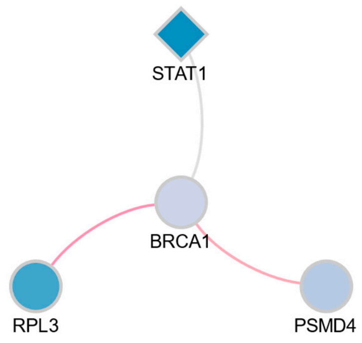

Figure S5. Protein-protein interaction of top 1 module gene (differentially expressed genes).

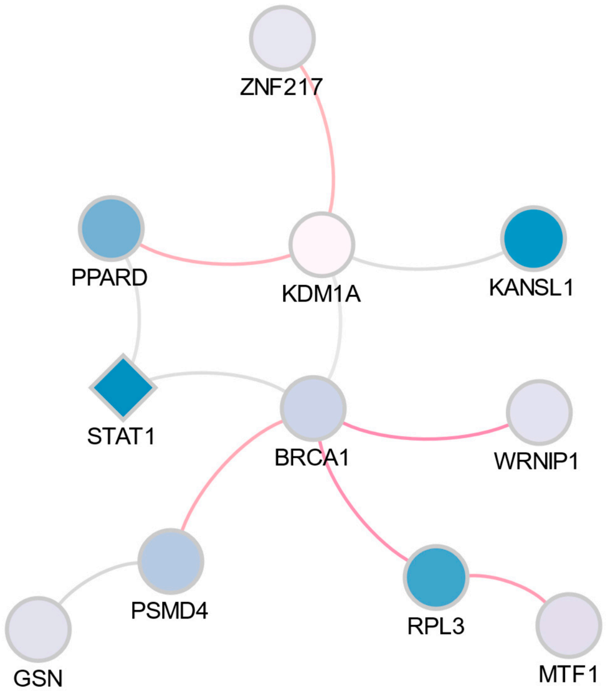

Figure S6. Protein-protein interaction of top 5 module gene (differentially expressed genes).

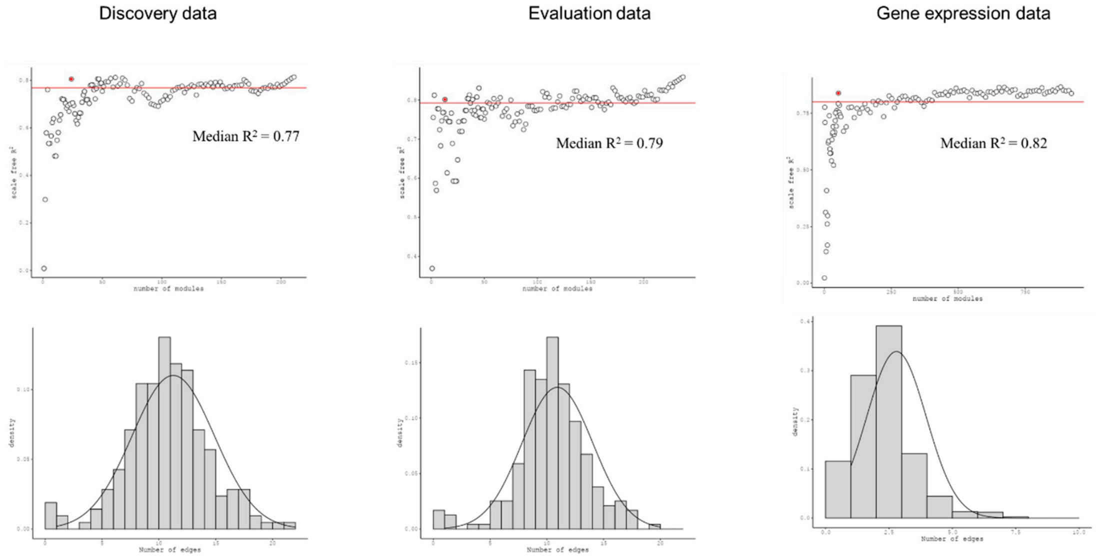

Figure S7. Characteristics of scale-free networks of resulting modules from dmGWAS and EW_dmGWAS analysis. Scatter plot of the scale-free index (R2) for networks constructed by top modules identified from dmGWAS and EW_dmGWAS analysis.

Density histogram shows the distribution of the number of edges of each gene within the gene network constructed by the TMGs of dmGWAS and EW_dmGWAS analysis.

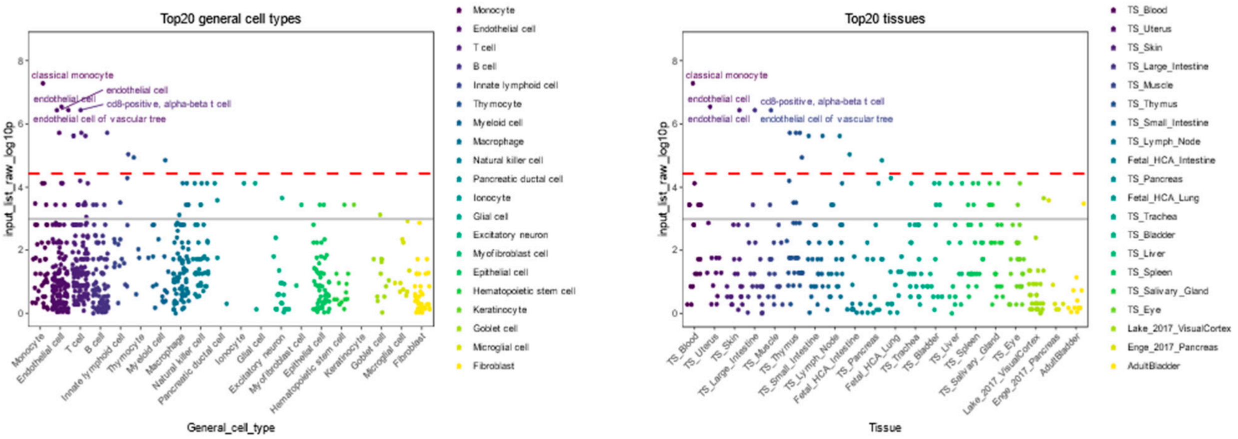

Figure S8. Cell-type and tissue analyses across top dmGWAS modules of discovery data.

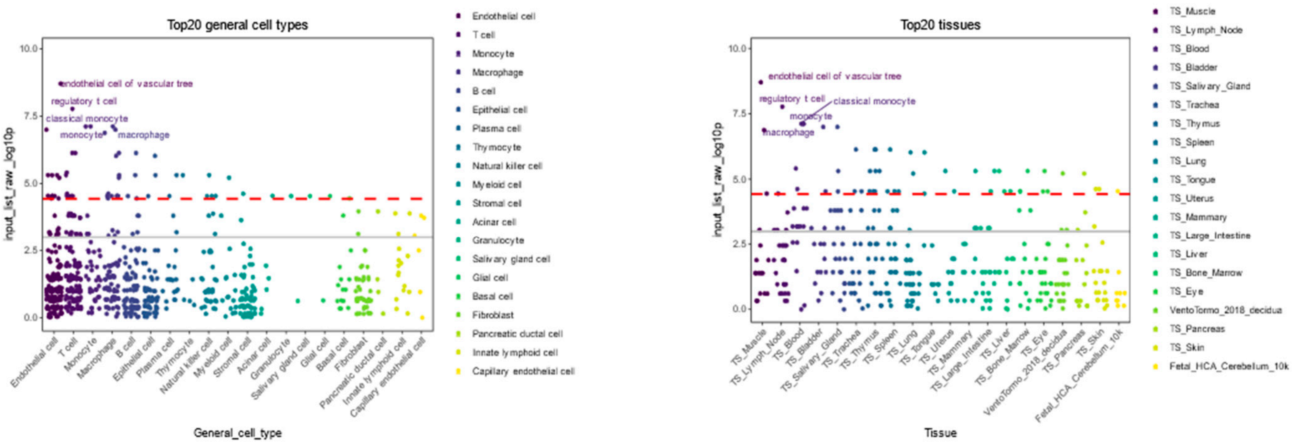

Figure S9. Cell-type and tissue analyses across top dmGWAS modules of evaluation data.

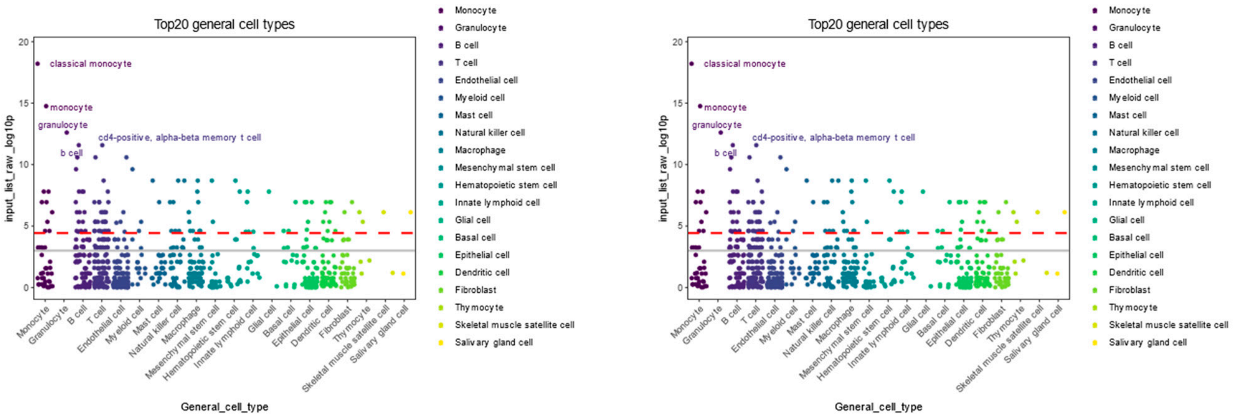

Figure S10. Cell-type and tissue analyses across top EW_dmGWAS modules.

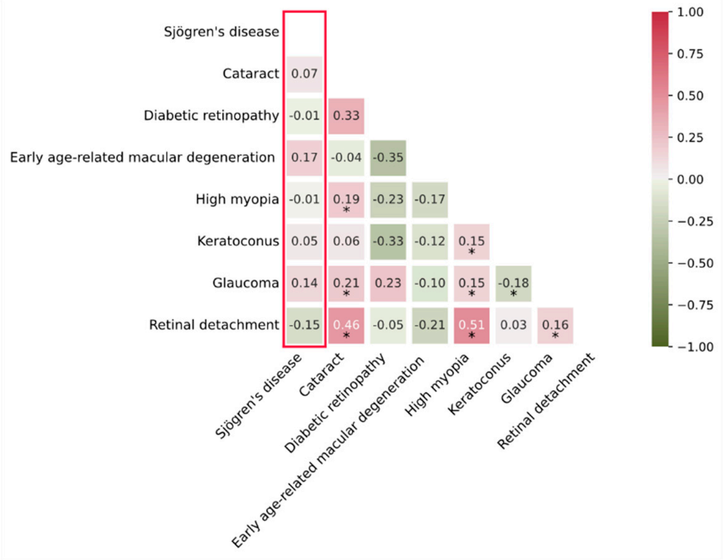

Figure S11. Heatmap of genetic correlation of SjD with ocular diseases

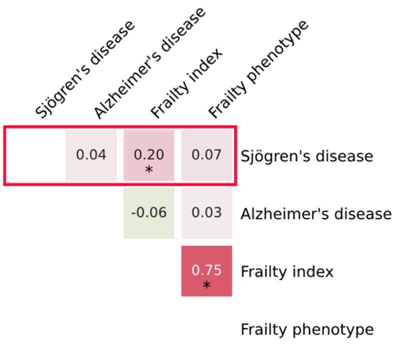

Figure S12. Heatmap of genetic correlation of SjD with aging diseases.

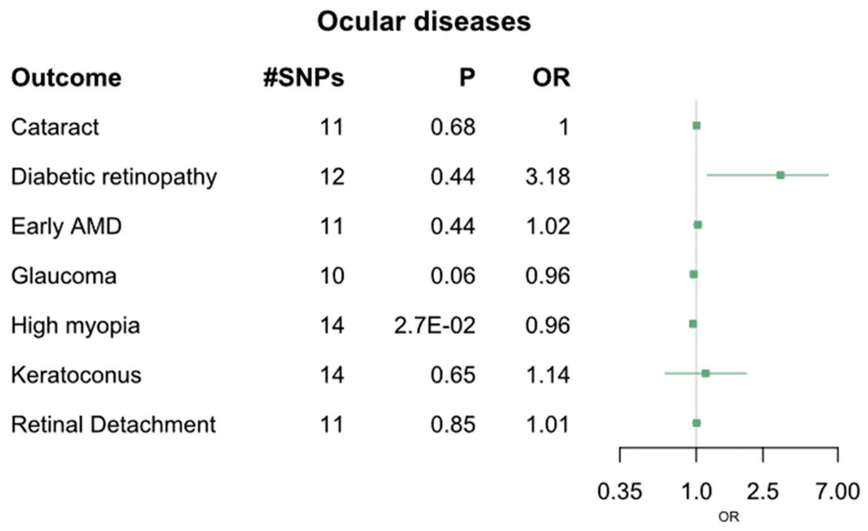

Figure S13. Mendelian randomization of SjD with ocular diseases

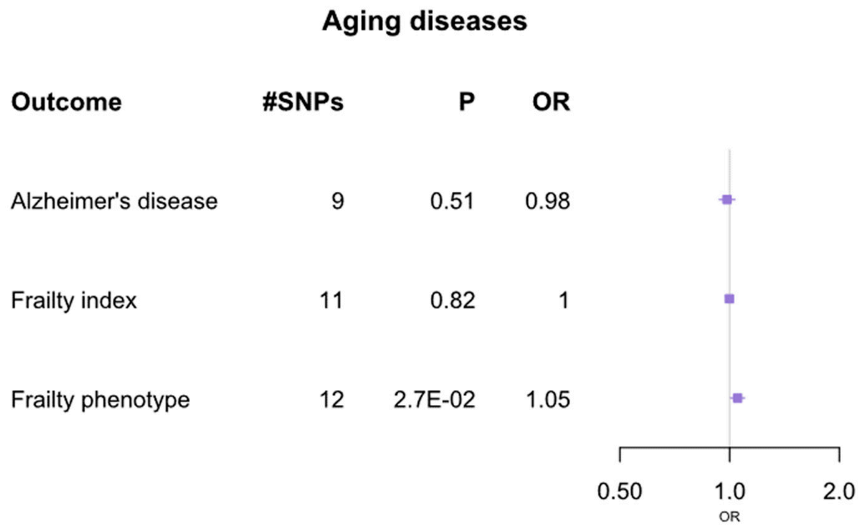

Figure S14. Mendelian randomization of SjD with aging diseases.
